# A case of episcleral tattooing – an emerging body modification trend

**DOI:** 10.1186/s12886-015-0095-y

**Published:** 2015-08-08

**Authors:** James Brodie, Husam El Galhud, Adam Bates

**Affiliations:** Ophthalmology Department, Maidstone Hospital, Hermitage Lane, Maidstone, Kent ME16 9QQ UK

## Abstract

**Background:**

In 2007 an article was published describing the first forays into the practice of episcleral tattooing. Currently only a handful of people worldwide have undergone this procedure, whereby a needle is used to inject dye under the bulbar conjunctiva. To date there have been no previous reports of the risks and complications of this emerging practice in the medical literature.

We present a case involving a complication that arose in one of the few people in Britain to have undergone episcleral tattooing for cosmetic purposes.

**Case presentation:**

A 43-year-old Caucasian man presented to the eye casualty clinic with red, lumpy conjunctivae bilaterally, having undergone episcleral tattooing 7 weeks previously. On examination there were 3 distinct areas of conjunctival swelling in each eye, representing a total of 6 injection sites. No other gross abnormalities were identified. The clinical picture remained unchanged 6 months on, apart from a degree of fading of the conjunctival dye. He will remain under our care to ensure that any further complications such as granulomatous inflammation are managed and documented.

**Conclusion:**

Episcleral tattooing is carried out by individuals with no medical training. The short-term complications reported so far include: headaches, severe photophobia, persistent foreign body sensation, and migration of ink staining. More serious short-term risks such as infection, globe penetration, and peri-ocular haemorrhage could occur. For now we can only speculate as to the long-term consequences, but these may include carcinogenic change or granulomatous inflammation. We feel that the potential risks of the procedure should be communicated more widely to those body modification practitioners undertaking it. This practice could result in more serious presentations to acute eye services in the future.

## Background

In 2007 the Body Modification Ezine published an article describing the first instances of episcleral tattooing [1]. At present only a handful of people worldwide have undergone episcleral tattooing, whereby dye is injected under the bulbar conjunctiva, for purely cosmetic purposes. There have been no previous reports of the risks and complications of this emerging practice in the medical literature.

We present a case involving a complication in one of the few people in Britain to have undergone episcleral tattooing.

## Case presentation

**Fig. 1:**
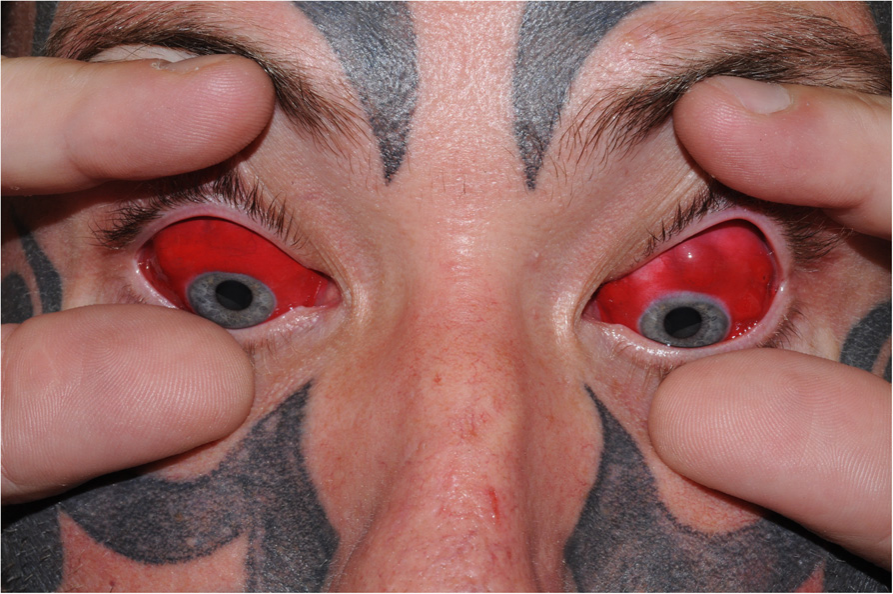
Bilateral episcleral tattoos

A 43-year-old man presented with bright red, lumpy conjunctivae bilaterally (see Fig. 1). 7 weeks previously he had undergone episcleral tattooing and his complaint was that the conjunctival lumps had not subsided. In this case a mixture of two dermal tattoo dyes (C.I Pigment Red 210 and C.I Pigment Blue 15) was injected under the conjunctiva at 3 sites in each eye. Visual acuity was 6/4 in both eyes and he reported no visual symptoms or discomfort.

**Fig. 2:**
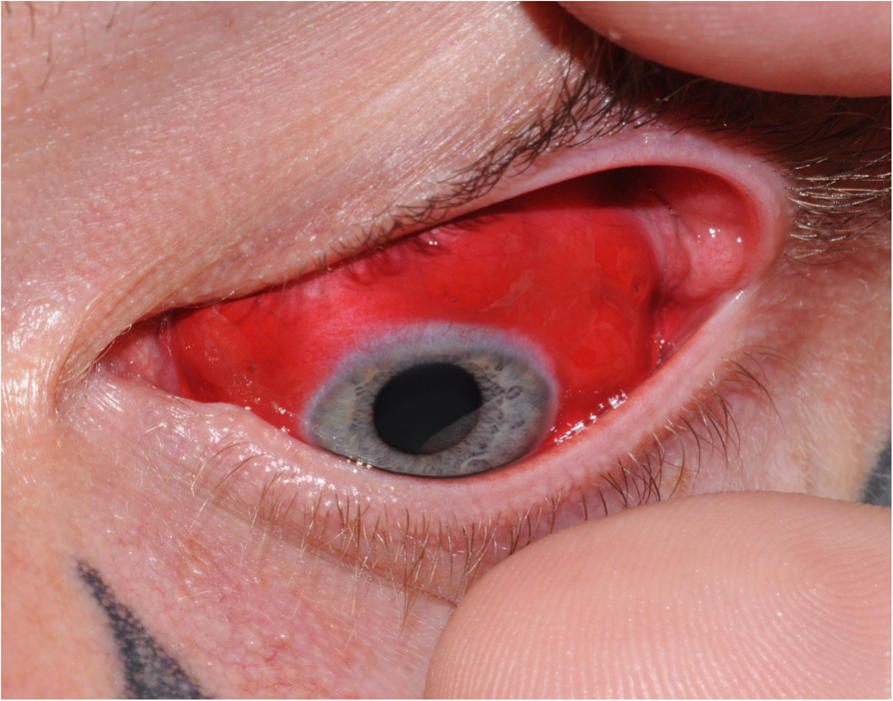
Left eye showing 3 distinct lumps at injection sites

**Fig. 3:**
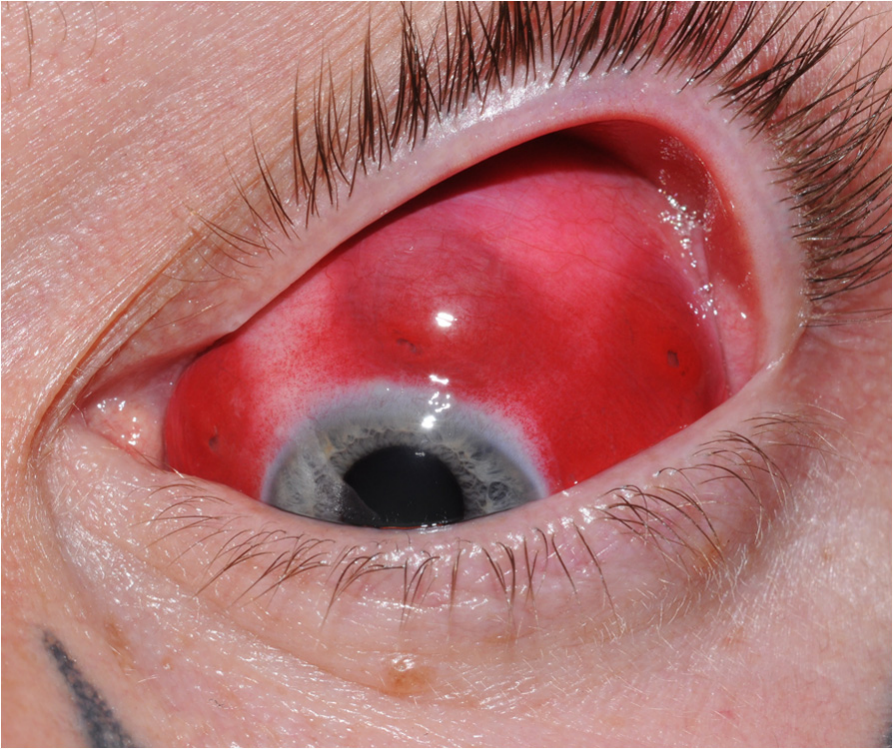
Left eye showing fading of conjunctival dye at 6 months follow up

On slit lamp examination there were 3 distinct areas of conjunctival swelling in each eye, representing a total of 6 injection sites (Fig. 2). Apart from this aesthetically poor, persistent lumpy appearance, no other gross abnormalities were identified. Fundal examination and intraocular pressure measurements were also normal. The clinical picture remained unchanged 6 months on, apart from a degree of fading of the conjunctival dye (Fig. 3). He will remain under our care to ensure that any further complications such as granulomatous inflammation are managed and documented.

## Conclusion

The number of body modification procedures carried out annually is increasing [2] and new trends are pushing the boundaries of “extreme” in many directions. Procedures such as episcleral tattooing are carried out by individuals with no medical training, who are taking it upon themselves to pioneer an untested practice through trial and error.

The short-term complications reported so far have included: headaches, severe photophobia, persistent foreign body sensation, and migration of ink staining to nearby tissues [3, 4]. Episcleral tattooing by a non-ophthalmic trained user without the use of a surgical microscope carries the risk of globe penetration, traumatic cataract, retinal detachment and endophthalmitis. In addition, tattoos carry the risk of blindness from tattoo-induced uveitis [5–8]. If distant skin tattoos can induce uveal sensitization to the dyes in the tattoo, placing the dye under the conjunctiva may result in more severe or more frequent uveitis. Finally, the presence of chronic granuloma can lead in the long term to scleral thinning or malignancy [9].

We feel that the potential risks of the procedure should be communicated more widely to those body modification practitioners undertaking it. This practice could result in more serious presentations to acute eye services in the coming years.

### Consent

Written informed consent was obtained from the patient for publication of this Case report and all of the accompanying images. A copy of the written consent is available for review by the Editor of this journal.
